# Götz Fabry: Medizindidaktik – Für eine kompetenzorientierte, praxisrelevante und wissenschaftlich fundierte Ausbildung

**DOI:** 10.3205/zma001638

**Published:** 2023-09-15

**Authors:** Angelika Homberg

**Affiliations:** 1 Medical Faculty Mannheim, Department of Medical Education Research, Heidelberg University, Mannheim, Germany

## Bibliographical details

Götz Fabry

**Medizindidakitik – Für eine kompetenzorientierte, praxisrelevante und wissenschaftlich fundierte Ausbildung**

Publisher: Hogrefe Verlag

Year of publication: 2023, 350 pages, price: € 59,95

ISBN: 978-3-456-85852-4

## Review

Medical didactics – do they exist at all? If so, what should they cover? As a specialized area of teaching, they could claim to be an educational science that pursues the central pedagogical questions about the *Why, What,* and *How* of teaching and learning in the respective subject, establishes references to general didactic principles, and encourages reflection on the individual and social relevance of the learning content and competencies to be taught. The question is: Does the author satisfy these requirements?

The first chapter addresses what kind of training future physicians actually need. The question is dealt with from different perspectives: from the university alumni point of view, from the field of patient care and from the field of higher education. Historical and international references are made, current studies are cited. The subject of medicine is placed in the context of scientific theory and the discourse of professionalization. The critical scrutiny of the author points out weaknesses in medical education, but leaves it to the reader to form his or her own opinions. The scope is initially broad, and ample impetus is given for reflection on the social relevance of successful medical education. 

The second chapter examines generally applicable concepts such as motivation, cognition and learning with regard to their significance for medical education. The author’s tone is not instructional, but rather he argues, substantiates and proves. If you want to teach, you have to understand how learning takes place – and this can vary greatly from individual to individual. So, learning strategies are differentiated, learning activity analyzed, clinical thinking categorized. In tables and using practical examples, concrete possibilities are shown how different activity levels can be addressed in teaching and how different teaching methods can support the development of professionalism in students. References to general didactics are made.

In the third chapter it all finally becomes concrete: “Lesson planning”. Or so I thought. In any case, the questions of *Why, What* and *How* are answered, again theoretically grounded, scientifically substantiated, and hermeneutically justified. Basic questions are first clarified, e.g., “What are competencies, really?” or “How can goals for medical education be defined?” and “What role does the National Competence Based Catalogue of Learning Objectives for Undergraduate Medical Education play here?” Particularly noteworthy are the learning goal taxonomies. Bloom’s taxonomy is familiar to (almost) every savvy educator, but did you know that it only covers the cognitive domain? And are you also aware of the learning goal categories for the affective and psychomotor domains? As annoying as it may be for teachers to first deal with competencies and learning objectives, this is then ultimately useful for further planning and instructional design, up to and including the development of a customized learning objective assessment. For the reader, the effort is worth it, even here. The real tips for teaching and learning are then finally found in chapter four “Teaching methods”. This chapter is sorted into “Formats for large groups”, “Learning in small groups”, and “Clinical teaching”, following the logic of the teaching formats at German medical schools. Here, the new edition benefits in particular from newly added topics and subchapters such as “Lecture Recordings”, “Flipped Classroom”, and “Feedback”. 

Chapters five “Examining: information and results monitoring” and six “Teaching evaluation” have also benefited from the revision of the earlier edition (2008). For example, the subchapter “Competence-oriented testing – what does that mean?” has been added. The question “What can/should be examined?” shows that we are looking far beyond the current state of affairs. Examinations are considered in their interaction at the curricular level, but are also scrutinized in detail, for example, with regard to their formal requirements and possible uses. The final chapter, as is often the case in teaching, is “Teaching evaluation”, which is mandatory under the German medical licensure regulations (ÄApprO, 2002), but the potential of which is often not fully exploited. Yes, valid statements on the quality of teaching can indeed be made. In return, however, the question is also asked “Can students judge the quality of teaching at all?” This weighing, contrasting, looking underneath the surface characterizes all of the chapters and invites more in-depth grappling with individual questions. The comprehensive subject index and effective organization of the material makes looking up specific information convenient.

Each individual chapter ends with a conclusion and, in addition to an extensive bibliography, also with recommendations for more in-depth reading. So-called toolboxes, 17 in total, include checklists, strategies and assessment sheets and provide a high level of practical relevance. 

The verdict? Medical didactics fulfill all of the requirements of a specialized area of teaching, and even more. The precision with which individual aspects are examined provides ample starting points for further development of teaching in research and pedagogy. Remarkable is the clarity and, at the same time, cautiousness with which criticism of the current state of education is exercised, constructively and concretely. The focus is on what has already been scientifically tested, what could be and what is feasible. It makes you want to do more.

If you are looking for a quick fix to brighten up your teaching, you will be disappointed. Good teaching is a complex undertaking with many influencing variables. Teachers, learners and researchers who want to look at teaching and learning in medicine in depth will find a real treasure chest here. It’s worth it.

## Competing interests

The author declares that she has no competing interests.

